# Maternal health insurance in Indonesia: assessment of a policy design

**DOI:** 10.1186/1471-2458-14-S1-P8

**Published:** 2014-01-29

**Authors:** Tiara Marthias, M Faozi Kurniawan, Likke Putri

**Affiliations:** 1 Universitas Gadjah Mada, Yogyakarta 55281, Indonesia

## Background

The main purposes of the Indonesian national insurance for maternal and delivery services were to: (1) increase antenatal and skilled birth attendance coverage, (2) increase neonatal and postnatal care, and postnatal family planning coverage, and (3) ensure effective and accountable health financing for MNCH.

However, there has been a growing concern on the implementation of the policy. Increased accessibility does not always translate to better health services and improved equity in health. The programme has also put dismaying burden on the health system, particularly for health providers. To ensure better programme implementation that could better achieve the original purposes, Jampersal health insurance should be scrutinised from the policy analysis perspective. This study aimed to analyse the design of the Jampersal policy in Indonesia and to provide suggestions for improvement for future implementation.

## Materials and methods

The study focused on analysing the design issues and the challenges, using a framework developed based on the purposes of the policy itself.

## Results

Key innovations of the policy included (1) the cover-all care package, (2) mandatory facility-based delivery, and (3) family planning programme. The innovations had the potential to increase MNCH care services accessibility. However, the limited budget ceiling may compromise high quality service. From the equity side, Jampersal is a non-targeting health insurance covering all women regardless of their socioeconomic status. Also, the policy did not address other barriers to health service, e.g. transportation costs for family members, and unclear financing scheme for caesarean section. Much of the funding relied on the sub-national management, which under the decentralisation, does not necessarily translate to clearer funding coverage.

## Conclusions

MNCH insurance policy could alleviate financial burden, and could be improved through strengthening the programme design and improving the implementation. The policy needs to be evidence-informed, so that it would be well targeted and achieve the main purposes of the programme itself.

